# Citrullinated histone H3 and procalcitonin in the diagnosis and predicting the outcome of septic shock

**DOI:** 10.1186/s12879-026-12802-z

**Published:** 2026-03-02

**Authors:** Nadia Mostafa Mohamed, Iman Hussein Shehata, Amr Hosny Hamza, Nermeen M. A. Abdallah

**Affiliations:** 1 Medical Microbiology and Immunology, Faculty of Medicine - Ain Shams University, Cairo, Egypt; 2 Anesthesia and Intensive Care Unit (ICU) Department. Faculty of Medicine - Ain Shams University, Cairo, Egypt

**Keywords:** Citrullinated histone H3, Septic shock, Sepsis, Biomarker, Procalcitonin, Neutrophil extracellular traps

## Abstract

Sepsis and septic shock stimulate a massive inflammatory response during which neutrophil extracellular traps (NETs) release citrullinated histone H3 (CitH3) into the circulation, leading to tissue damage, coagulopathy, and organ failure. This study aimed to assess serum levels of CitH3 in patients with septic and non-septic shock in intensive care units and to study their correlation to septic shock severity. The study was conducted at Ain Shams University Hospitals and included 72 adult participants: 24 with septic shock, 24 with non-septic shock, and 24 healthy controls. Serum CitH3and procalcitonin (PCT) levels were measured using ELISA, and blood cultures were performed for septic shock patients. Median CitH3 levels were 5.58 ng/mL in healthy controls, 44.38 ng/mL in non-septic shock, and 198.7 ng/mL in septic shock patients (*p* < 0.001). CitH3 strongly correlated with SOFA scores (Non-septic: *r* = 0.793; Septic: *r* = 0.786) and ICU stay in septic patients (*r* = 0.477, *p* = 0.019). ROC analysis showed CitH3 performed better than PCT in distinguishing septic from non-septic shock patients (AUC 0.946 vs 0.747, *p* = 0.012) and from controls (AUC 0.991 vs 0.853, *p* = 0.017). Direct comparison also showed that CitH3 had superior predictive performance for mechanical ventilation (*p* = 0.038). CitH3 could be considered a reliable biomarker of septic shock. It can be regarded as a valuable tool for predicting prognosis and severity of sepsis. CitH3 could be a promising candidate for routine integration into sepsis management protocols.

## Clinical trial number

Not applicable.

## Introduction

Sepsis is considered a severe and potentially life-threatening condition caused by an uncontrolled immune response to infection. Progression of this dysregulated response can lead to septic shock, where worsening inflammation, tissue hypoperfusion, and, eventually, organ failure can occur [1]. Sepsis is a leading cause of death worldwide, and in low- and middle-income countries, it shows higher documented rates mainly due to delayed diagnosis and treatment. Early recognition of progression to septic shock is a vital step in reducing mortality. Laboratory markers play a significant role in diagnosing, monitoring, and guiding treatment in patients with sepsis and septic shock [2].

Neutrophils play a crucial role in the pathogenesis of sepsis. Neutrophils migrate to infection sites and create the neutrophil extracellular traps (NETs) through a process known as NETosis. NETs are made of histones, decondensed DNA fibers, neutrophil elastase, myeloperoxidase, and other proteins from neutrophil organelles [3]. NET formation requires histone H3 citrullination; it is mainly produced through Protein Arginine Deiminase 4 (PAD4)-mediated posttranslational modification (citrullination) of histone H3 [4, 5]. Neutrophils are the dominant source of extracellular CitH3 in sepsis [6]. Beyond neutrophils, monocytes, macrophages, eosinophils, mast cells, and plasmacytoid dendritic cells can all release CitH3 [7]. Although the prominent role of NETs in the containment and destruction of pathogens, overproduction of NETs can lead to endothelial damage, trigger coagulation pathways, encourage microthrombosis, and worsen multi-organ dysfunction [8].

Citrullinated histones, especially citrullinated histone 3 (CitH3), are emerging as a highly specific marker of sepsis, particularly in its most severe forms. Several studies have identified CitH3 as a promising biomarker of NET formation. CitH3 levels in septic patients are elevated, and they are closely associated with the severity of illness, organ dysfunction, and death. Evidence from experimental and clinical studies suggests that infectious triggers (bacteria, viruses) induce stronger PAD4 activation, leading to higher CitH3 levels. Circulating CitH3 increases inflammation, attracts and activates leukocytes, and sustains NETosis in a positive feedback loop [6, 9]. To emphasize the role in sepsis, Anti-citrullinated histone antibodies targeting CitH3 have shown a promising therapeutic potential in sepsis by neutralizing the harmful effects of extracellular histones released during NETosis [5].

Besides sepsis, non-infectious stimuli (trauma, burns, sterile inflammation) produce weaker activation and lower CitH3 levels, which may contribute to tissue injury and thrombosis [9]. Shock, even when not caused by infection, triggers a systemic inflammatory response that activates neutrophils and releases citrullinated histone. Additionally, damage-associated molecular patterns (DAMPs) from injured tissues can mimic infection signals, resulting in NETosis [10]. Therefore, elevated CitH3 can reflect severe cellular injury and systemic inflammatory response, even in the absence of infection.

Till now, no single ideal marker for diagnosing and predicting sepsis progression has been identified, but many help identify critically ill patients who need close monitoring, enabling prompt treatment. Of these markers, CRP (C-reactive protein), PCT, and lactate were examined. [11]. However, due to their limited specificity and sensitivity, there is a need for more reliable markers. This study aimed to assess serum levels of CitH3 in patients with septic and non-septic shock in intensive care units and to study its correlation with septic shock severity.

## Methods

This case-control study was conducted at Ain Shams University Hospitals from December 2024 to May 2025, involving 48 hospitalized adult patients in intensive care units (ICUs) (24 diagnosed with septic shock and 24 diagnosed with non-septic shock), as well as 24 non-hospitalized, healthy individuals. Based on Sepsis-3 criteria, septic shock patients were included if they had a suspected infection and required vasopressors to maintain a mean arterial pressure of ≥ 65 mmHg and a serum lactate level of ≥ 2 mmol/L despite adequate fluid resuscitation. Patients with disseminated malignant disease, pregnancy, or in immunosuppressive status were excluded. Immunosuppression was defined as patients with an absolute neutrophil count (ANC) < 1500/µL, receiving chemotherapy, receiving long-term corticosteroid therapy, receiving other immunosuppressive treatments, or having acquired immunodeficiency syndrome.

Non-septic shock patients included those with hypovolemic shock, hemorrhagic shock, cardiogenic shock, and obstructive shock (pulmonary embolism). Normal healthy individuals who were blood donors with no chronic medical conditions, not taking any medications, and symptom-free at the time of enrolment were included.

Informed consent was obtained from all participants. The study was approved by the Ethics Committee of Scientific Research of the Faculty of Medicine, Ain Shams University, under the code No: (FMASU MS 89/2025)

Full history was taken from the patients, including demographic and clinical data, Sequential Organ Failure Assessment (SOFA) score, exposure to invasive devices, primary site of infection, duration of mechanical ventilation, length of ICU stay, length of hospital stay, and 28-day mortality were recorded for patient of septic shock, beside the results of the complete blood count (CBC), CRP level, and serum lactate.

### Collections of blood samples

Blood samples were collected at the time of patient enrollment, within the first 24 hours of septic shock diagnosis. For septic shock patients, two peripheral venous blood samples (10 ml each) were collected from different body sites, spaced at least an hour apart. Blood cultures were performed using the BacT/ALERT 3D 60 automated microbial detection system (bioMérieux, Marcy-l’Étoile, France), following the manufacturer’s instructions. A maximum of 5 mL of the sample was inoculated into a BacT/Alert aerobic resin bottle (FA Plus) and incubated in the BacT/ALERT® 3D system for up to 5 days or until a positive signal was detected. Bacterial identification and confirmation were subsequently performed using the VITEK® 2 Compact 15 system (bioMérieux, Marcy-l’Étoile, France)

Blood samples of 2 mL were collected from all study participants in tubes without additives, kept at room temperature for 10–20 minutes to allow clotting, and then centrifuged at 2000–3000 RPM for 20 minutes. The supernatant was collected. and immediately frozen in sterile tubes at −80 °C until performing the ELISA assay to measure serum levels of CitH3 and procalcitonin. using the Human Citrullinated Histone H3 ELISA Kit and the Human Procalcitonin ELISA Kit from Bioassay, England/China, following the manufacturer’s instructions.

### Sample size calculation

Sample size calculation was performed using PASS version 15 software, setting power at 90% and alpha error at 0.01. Based on data reported by Tian et al. (2021) [6], a sample size of 20 participants per group was required to detect a statistically significant difference between the three groups using one-way ANOVA. Accordingly, 24 participants were recruited per group.

### Data management and analysis

Data were analyzed using the Statistical Package for the Social Sciences (SPSS, version 25). The Kolmogorov-Smirnov test was employed to assess the normality of continuous variables. Descriptive statistics were presented as means and standard deviations for normally distributed quantitative data, and as medians with interquartile ranges (IQR) for non-normally distributed data. Categorical variables were summarized using frequencies and percentages. Bivariate analyses were conducted using analysis of variance (ANOVA), the Kruskal-Wallis test, independent samples t-test, Mann-Whitney U test, Chi-square test, and Pearson correlation, as appropriate.

The area under the receiver operating characteristic curve (AUC-ROC) was calculated to evaluate the diagnostic and predictive performance of PCT and CitH3 in differentiating between septic shock patients and healthy controls, as well as between septic shock and non-septic shock patients. Additionally, their ability to predict mortality and the need for mechanical ventilation was assessed. Sensitivity, specificity, positive predictive value (PPV), and negative predictive value (NPV) were also computed. A p-value of < 0.05 was considered statistically significant. A direct statistical comparison between the ROC curves of CitH3 and PCT was done using a Z-test for correlated AUCs.

## Results

### Patients characteristics

**Fig. 1:**
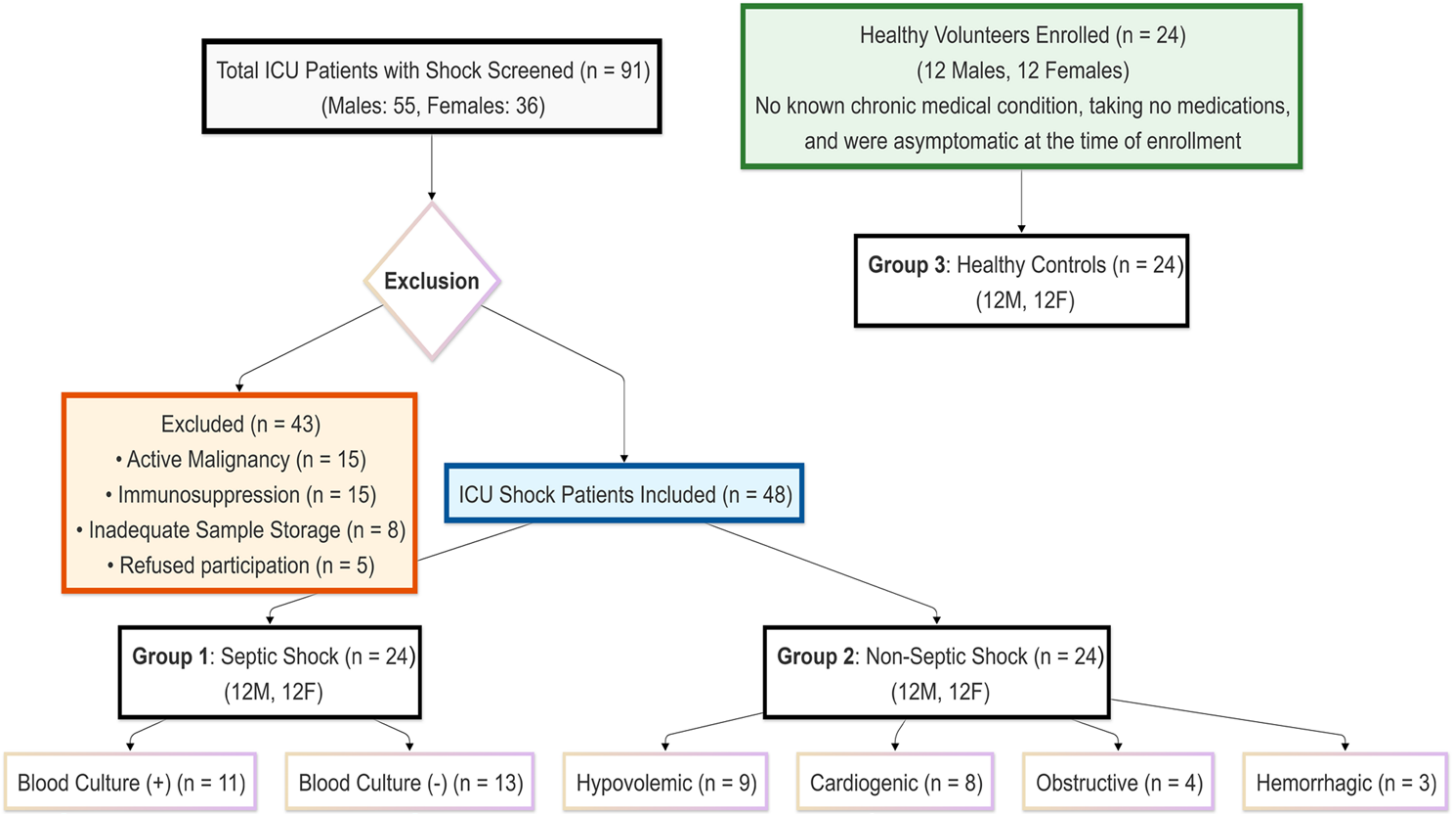
Flowchart of study participants

This study included 72 adults (aged 18 years or older) divided into three groups. Figure 1 presents a flowchart of the patients included in the study. Demographic data for all participants and clinical and laboratory data for patients are summarized in Table 1.

**Table 1:** Comparison between healthy controls, non-septic shock controls, and septic shock cases regarding demographic data, clinical, and laboratory parameters

		Healthy controls	Non-septic shock controls	Septic shock cases	P value
		**No**. = 24	No. = 24	No. = 24	
Age (years)	Mean (SD)	43 (15)	63 (14)	58 (18)	F < 0.001*
	Range	22–72	27–81	26–84	
Sex	Male	12(50%)	12(50%)	12(50%)	—
	Female	12(50%)	12(50%)	12(50%)	
CRP (mg/l)	Median (IQR)	-	104(47- 177)	208(149.2 - 296.8)	*U* > 0.001*
	Range	-	(6.9- 417.6)	(97- 407)	
SOFA	Mean (SD)	-	8 (3)	12 (3)	*T* > 0.001*
	Range	-	3- 13	7- 17	
Lactate (mmol/l)	Mean (SD)	-	3.1 (0.9)	3.2 (1.2)	*T* > 0.001*
	Range	-	2.1- 5.4	2.1- 7.8	
TLC (10^3/µl)	Mean (SD)	-	10.7 (3)	19.9 (12)	*T* = 0.002*
	Range	-	6.4- 17.4	2.2- 64	
ANC(10^3/µl)	Mean (SD)		7.24 (3.51)	16.21 (10.91)	*T* > 0.001*
	Range		2.9–15.9	1.7–55	
Length of ICU stay(days)	Mean (SD)	-	7(4)	9(4)	*T* = 0.08
	Range	-	1–13	3–18	
Mechanical ventilation	No	-	12(50%)	8(33.3%)	C = 0.312
	Yes	-	12(50%)	16(66.7%)	
28-day mortality	Survive	-	13(54.2%)	8(33.3%)	C = 0.129
	Died	-	11(45.8%)	16(66.7%)	
Outcomes among survivors	discharged	-	3(23.2%)	2(25%)	C = 0.912
	improved	-	6(46.2%)	3(37.5%)	
	deteriorated	-	4(30.6%)	3(37.5%)	

*Sig p-value, test of significance, One-way ANOVA(F), Independent samples t-test (T), Mann-Whitney test (U), Chi-square (C)

When comparing septic and non-septic cases, the SOFA score, CRP levels, lactate levels, total leukocyte count (TLC), and absolute neutrophil count (ANC) were significantly higher in the septic group than in the non-septic group. The septic group patients had more extended ICU stay, higher rates of need for mechanical ventilation, and higher 28-day mortality rates compared to non-septic ones; this difference was not statistically significant

### Results of blood culture

**Fig. 2:**
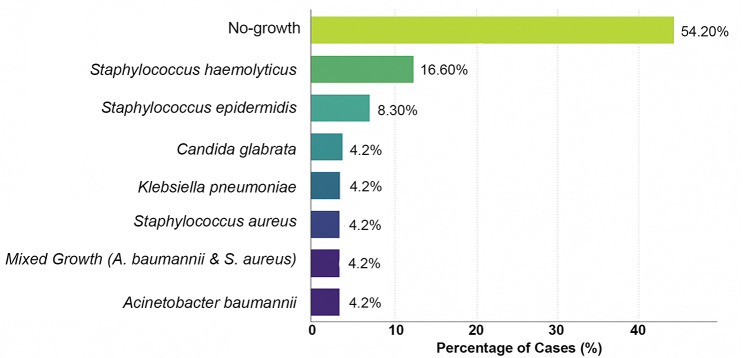
Results of blood culture of septic shock cases

Blood cultures were negative in 13 patients (54.2%) and positive in 11 patients (45.8%). Isolated organisms included *Staphylococcus haemolyticus, Staphylococcus epidermidis, Acinetobacter baumannii, Klebsiella pneumoniae, Staphylococcus aureus,* mixed infection of *Acinetobacter baumannii* with *Staphylococcus aureus*, and *Candida glabrata*. Fig. 2.

### Results of procalcitonin and citrullinated histone 3 ELISA tests

**Fig. 3:**
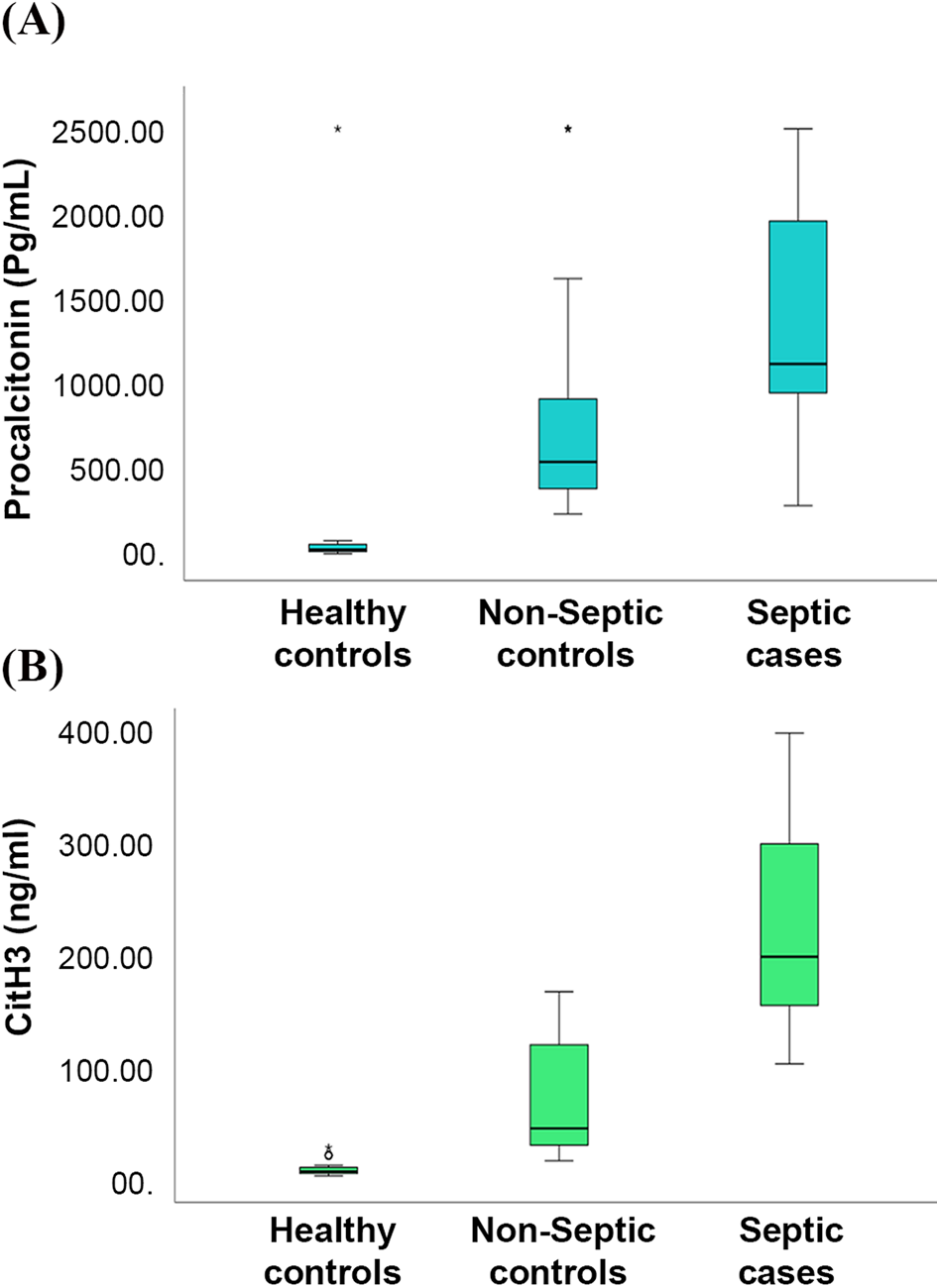
Comparison between healthy controls, non-septic shock controls, and septic shock cases regarding procalcitonin (**A**), CitH3 (**B**)

The median PCT level in the control group was 35.64 pg/mL, which increased to 528.3 pg/mL in non-septic shock controls and then to 1078.5 pg/mL in septic shock cases. This upward trend was statistically significant (p-value < 0.001). Fig. 3A.

Citrullinated histone 3 levels also followed a similar pattern, with the control group showing a median of 5.58 ng/mL, non-septic cases rising to 44.38 ng/mL, and septic cases reaching a median of 198.7 ng/mL. Again, the differences were highly significant (p-value < 0.001). Fig. 3B.

CitH3 showed highly significant positive correlations with SOFA scores in both patient groups (Non-septic: *r* = 0.793; Septic: *r* = 0.786), and CitH3 correlated with longer ICU stays in septic patients (*r* = 0.477, *p* = 0.019), Table 2.

**Table 2:** Correlation analysis between PCT, CitH3 levels, and clinical parameters in non-septic shock controls versus septic shock cases

	PCT (pg/mL)				CitH3 (ng/ml)			
	Non-septic shock controls		Septic shock cases		Non-septic shock controls		Septic shock cases	
	r	P-value	r	P-value	r	P-value	r	P-value
CitH3 (ng/ml)	−0.134	0.542	−0.326	0.120	-	–	-	-
PCT (pg/mL)	-	-	-	-	−0.123	0.542	−0.326	0.120
SOFA score	−0.023	0.916	−0.249	0.240	0.793	< 0.001*	0.786	< 0.001*
CRP (mg/l)	0.098	0.657	0.304	0.148	0.049	0.824	−0.073	0.733
Lactate	−0.081	0.715	−0.096	0.654	0.123	0.591	0.128	0.550
TLC (10^3/µl)	0.092	0.677	0.042	0.846	0.056	0.800	0.040	0.852
ANC(10^3/µl)	−0.92	0.670	0.071	0.740	−0.187	0.381	−0.326	0.120
Length of ICU stay	0.054	0.805	−0.111	0.605	0.232	0.287	0.477	0.019*

Test of significance, Pearson correlation (r)

### Diagnostic accuracy of PCT and CitH3 to differentiate septic shock patients from non-septic shock patients and healthy controls

**Fig. 4:**
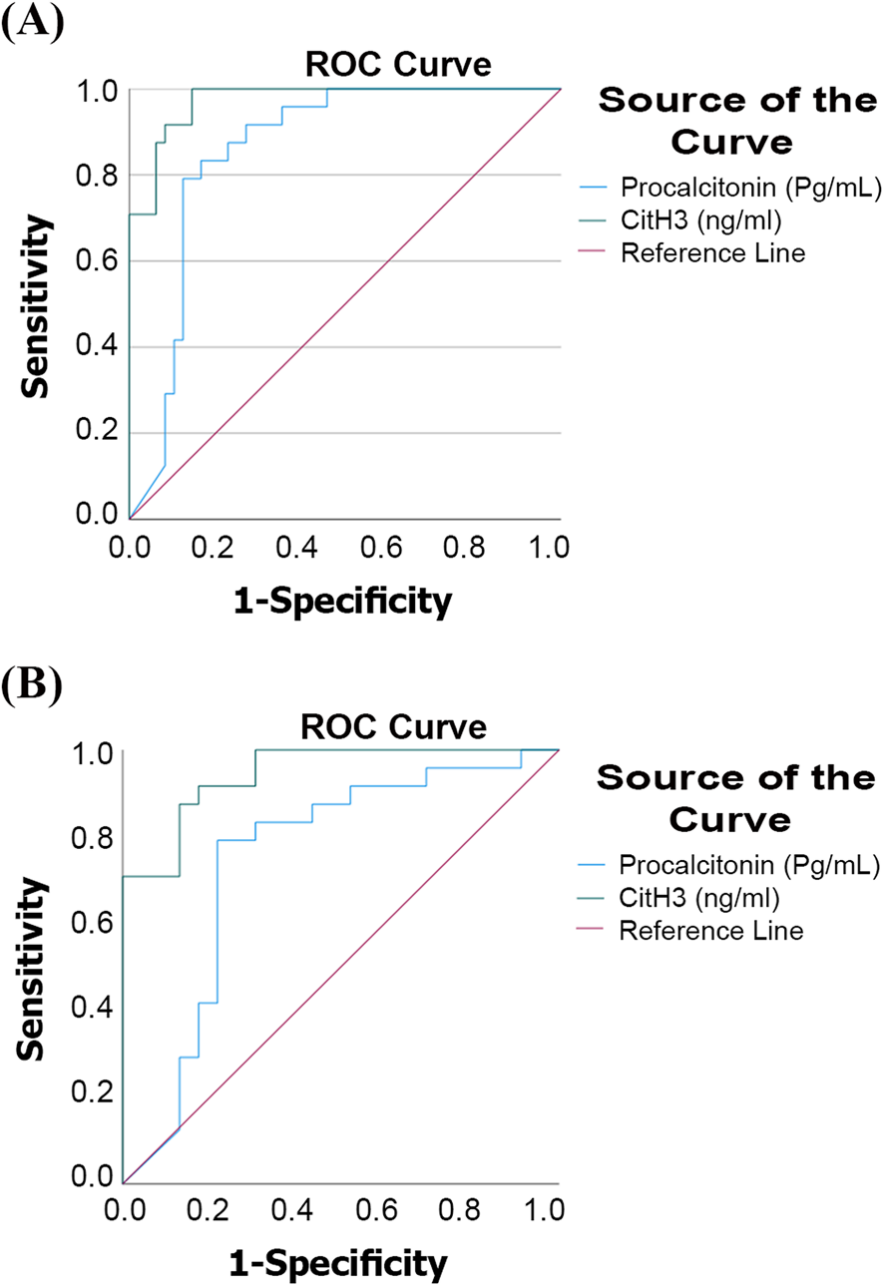
(**A**): ROC curve for PCT and CitH3 in differentiating septic shock cases from healthy controls (**B**): ROC curve for PCT and CitH3 in differentiating septic shock cases from non-septic shock controls

Based on ROC curves, the diagnostic value of CitH3 and PCT for differentiating septic shock from non-septic shock patients, as well as for differentiating septic shock cases from healthy controls, was evaluated. Fig. 4.

For differentiating septic shock from non-septic shock patients, PCT had an AUC of 0.747 (*p* = 0.004), with 79.17% sensitivity and 78.26% specificity at a cut-off value of 891.8 pg/mL. The PPV was 79.17%, and the NPV was 78.26%. When septic shock patients were compared with healthy controls, PCT showed an AUC of 0.853 (*p* < 0.001), with 96% sensitivity, 100% specificity, 100% PPV, and 96% NPV at a cut-off value of 282.2 pg/mL.

For CitH3, differentiating septic shock from non-septic shock yielded an AUC of 0.946 (*p* < 0.001) at a cut-off value of 130.7 ng/mL, with 91.67% sensitivity, 82.61% specificity, 84.62% PPV, and 90.48% NPV. When comparing septic shock patients with healthy controls, CitH3 showed an AUC of 0.991 (*p* < 0.001), with 99% sensitivity, 100% specificity, 100% PPV, and 99% NPV at a cut-off value of 27 ng/mL.

The comparative ROC analysis showed a statistically significant difference between CitH3 and PCT in differentiating septic shock patients from healthy controls (*p* = 0.017) and from non-septic shock controls (*p* = 0.012).

### CitH3 and PCT for predicting 28‑day mortality and need for mechanical ventilation

The ROC curve analysis for predicting 28-day mortality showed an area under the curve (AUC) of 0.733 (*p* = 0.007) for CitH3, which displayed 92.59% sensitivity, 55% specificity, 73.53% positive predictive value, and 84.62% negative predictive value at a cutoff value of 48.1 ng/ml.

**Fig. 5:**
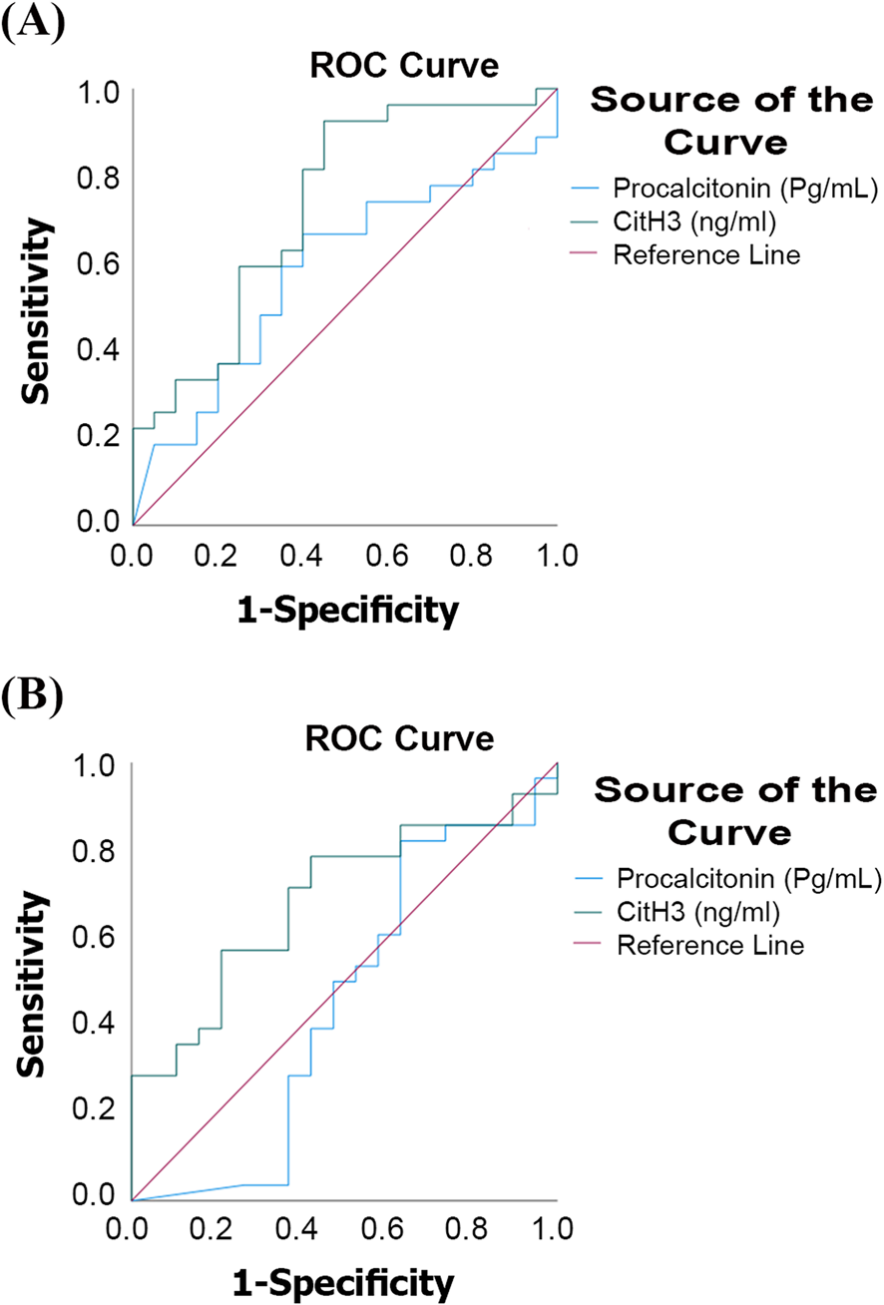
(**A**): ROC curve of PCT and CitH3 for predicting mortality. **B**: ROC curve of PCT and CitH3 for predicting mechanical ventilation

For PCT (AUC) of 0.586 (*p* = 0.317), and displayed 66.67% sensitivity, 60% specificity, 69.23%positive predictive value, and 57.14% negative predictive value at a cutoff value of 872.4 pg/ml. Fig. 5A.

The comparative ROC analysis showed no statistically significant difference between CitH3 and PCT for mortality prediction in septic and non-septic shock patients (*p* = 0.180).

For predicting the need for mechanical ventilation, the ROC curve analysis for CitH3 (AUC) of 0.686 (*p* = 0.032) and displayed 78.57% sensitivity, 57.89% specificity, 73.33% positive predictive value, 64.71% negative predictive value at a cutoff value of 117.5 ng/ml

For PCT, the area under the curve (AUC) of 0.448 (*p* = 0.552), with 82.14%sensitivity, 36.84% specificity, 65.71%positive predictive value, 58.33%% negative predictive value at a cutoff value of 510 pg/ml Fig. 5B.

The comparative ROC analysis showed a statistically significant difference in predicting the need for mechanical ventilation (*p* = 0.038).

## Discussion

CitH3 is strongly linked to NET formation, which is a critical process in the development of sepsis. The specificity of CitH3 for infection-driven immune responses made it more effective than traditional inflammatory markers in distinguishing septic shock from non-septic shock patients [12]. The results of the present study showed a consistent elevation of both PCT and CitH3 markers, corresponding to the severity of the clinical condition, from health to non-septic shock, and finally to septic shock. This is in good agreement with recent research that supports the diagnostic utility of these biomarkers in differentiating sepsis.

Median serum levels of CitH3 in this study were 198.7 ng/mL in septic shock patients, which were significantly higher than those of non-septic shock patients and healthy controls (*p* < 0.001). These findings were consistent with those reported by Tian et al. (2021), who observed that CitH3 concentration in septic shock patients was 101.5 pg/mL, while levels in non-infectious shock remained around 34 pg/mL. The even higher CitH3 levels observed in the present study may reflect earlier blood sampling during the initial hyper-inflammatory phase of sepsis, when CitH3 peaks, or a higher burden of underlying comorbidities (e.g., diabetes, chronic kidney disease) that are known to exacerbate sepsis severity [6, 13]. Beltrán-García et al. (2021) in their pilot ICU study comparing sepsis, septic shock, and controls, showed that plasma CitH3 was clearly elevated in septic shock patients, showing a mean of 15.36 ng/ml [14] A study comparing the biomarkers of NET formation in septic shock and severe COVID-19 patients reported CitH3 levels of 2.1–4.1 ng/mL [15]. While in cases of pediatric sepsis, at the time of diagnosis, CitH3 values ranged from 1080–2110 ng/mL with a cut-off of 1200 ng/mL (AUC 0.692) [16]. Age-related variations in immune response, NETosis capacity, patient selection, and methodological differences are likely to explain differences in CitH3 levels across these studies.

The level of CitH3 observed in this study showed a statistically significant positive correlation with SOFA scores in both non-septic and septic groups, suggesting that elevated CitH3 is associated with the degree of organ dysfunction, regardless of sepsis status. Furthermore, CitH3 levels were positively correlated with prolonged ICU stay in septic patients. This relationship may be explained by the role of CitH3 acting as a DAMP, activating Toll-like receptor 2, Ca^2 +^ ‑dependent PAD2, and further CitH3 production, driving a vicious cycle of NETosis, pyroptosis, and tissue injury [17]. Furthermore, CitH3 contributes to endothelial barrier dysfunction and adherens junction opening, increasing microvascular permeability and contributing to multi-organ failure [18]. These findings are consistent with previous research. For instance, Tian et al. (2021) demonstrated that CitH3 levels distinguished between septic and non-septic shock patients and correlated with the SOFA score. Similarly, Pan et al. (2021) reported that CitH3 was elevated in septic patients with acute pancreatitis and positively correlated with SOFA scores. Additionally, the septic group had a longer stay in the ICU. Collectively, our findings support CitH3‘s prognostic significance for predicting disease severity and clinical course in critical care [6, 19].

CitH3 and PCT were evaluated as diagnostic biomarkers in two settings to differentiate septic shock from both healthy controls and non-septic shock subjects, with CitH3 performing better than PCT in both settings. CitH3 demonstrated promising diagnostic accuracy (cut-off: 27 ng/mL; AUC: 0.991; sensitivity: 99%; specificity: 100%) compared with healthy individuals. PCT showed lower performance in the same context (AUC: 0.853). CitH3 also maintained better accuracy (cut-off: 130.7 ng/mL; AUC: 0.946) than PCT (AUC: 0.747) in distinguishing between septic and non-septic shock. Direct comparison of the ROC curve shows that CitH3 outperformed PCT in diagnostic precision (*p* = 0.017) (*p* = 0.012).

These results are notably higher than those reported by Tian et al. (2021), who observed AUCs of 0.91 for CitH3 and 0.43 for PCT when comparing septic shock patients with healthy individuals, and AUCs of 0.76 for CitH3 and 0.57 for PCT when comparing septic shock patients with non-septic shock patients. The better results in this study might be due to differences in patient selection criteria, timing of sample collection [20], and different testing methods [21].

Compared to the study by Pan et al. (2021), which reported CitH3 with an AUC of 0.94 and 0.93 for differentiating septic acute pancreatitis patients from healthy controls and from non-septic patients, the present study showed even higher diagnostic accuracy for differentiating septic shock from healthy controls (AUC: 0.991) and from non-septic shock (AUC: 0.946).

CitH3 is closely associated with NET formation, a crucial step in the progression of sepsis. In septic shock, overactivation of neutrophils leads to extensive NET release, resulting in elevated circulating CitH3 levels that reflect organ dysfunction, immune dysregulation, and endothelial injury. Supporting this mechanistic role, animal studies have shown that in murine models of lipopolysaccharide(LPS)-induced endotoxic shock, circulating CitH3 was significantly increased after lipopolysaccharide injection (1.18 ± 0.3 ng/mL vs. 0 ± 0 ng/mL, *p* = 0.0025), whereas CitH3 remained undetectable after hemorrhagic shock [22]. Furthermore, inhibition of PAD2/PAD4 in these models reduced CitH3 levels, attenuated NET formation, and improved survival, highlighting the causal role of CitH3 in sepsis pathophysiology [23]. These findings support the emerging role of CitH3 as a highly specific marker of sepsis-specific immune dysregulation and NET formation, processes that are minimally activated in non-infectious shock states, thereby enhancing its discriminatory power. The improved performance of CitH3 may be beneficial in ICU settings, where rapid differentiation between septic and non-septic shock can help guide crucial management decisions, such as the prompt administration of antibiotics and vasopressors. [24, 25].

Regarding mortality prediction, the present study demonstrates that CitH3 is a more reliable biomarker than PCT in predicting mortality in septic shock patients compared to non-septic shock patients, with a higher AUC (0.733 vs. 0.586), sensitivity (92.6% vs. 66.7%), and NPV (84.6%). However, there was no statistically significant difference between CitH3 and PCT in predicting mortality among septic and non-septic shock patients (*p* = 0.180). Previous studies on animal models showed that early high CitH3 levels in murine LPS shock occurred only in the lethal high‑dose group and were closely associated with 100% mortality. In contrast, low‑dose animals group had undetectable CitH3 and a 90% survival rate [26]. These findings are supported by earlier human research studies, including Tian et al. (2021), which linked elevated levels of CitH3 to 90-day mortality in septic shock patients. A key distinction is that the current analysis assessed CitH3’s predictive performance across the entire shock patients, rather than in septic shock alone. This broader approach may represent a more clinically relevant scenario in which patients often present with undifferentiated or mixed forms of shock. Additionally, unlike Tian et al. (2021), who assessed long-term mortality outcomes, our study focused on early in-hospital mortality. Beltrán-García et al. (2021) demonstrated that non‑survivors had higher CitH3 (median 19.0 vs 14.1 ng/mL). A cutoff of 14.81 ng/mL yielded AUC 0.897, sensitivity 83.3%, and specificity 81.0% for ICU mortality prediction [14]. Another study by Pan et al. (2021) confirmed CitH3 as an independent predictor of mortality in patients with septic acute pancreatitis. While their research was limited to a specific infection-related condition, the present study included patients with a wide range of shock etiologies, including non-infectious causes. This adds strength to the argument that CitH3 is not only a sepsis-specific marker but also a general marker of poor outcome in shock states [6, 19].

According to the current study, CitH3 is a more accurate biomarker than PCT for determining whether septic and non-septic shock patients may require mechanical ventilation. CitH3 demonstrated a statistically significant AUC of 0.686, with 78.6% sensitivity and 57.9% specificity at a cutoff value of 117.5 ng/mL. PCT had a non-significant AUC of 0.448 and somewhat greater sensitivity (82.1%), but it has limited predictive value due to its low specificity (36.8%). The ROC analysis comparing CitH3 and PCT revealed a statistically significant difference in predicting the need for mechanical ventilation (*p* = 0.038).

These results are supported by emerging data showing a direct link between CitH3 and lung injury and gas exchange impairment. Tian et al. (2021) reported elevated CitH3 levels in patients with sepsis-induced acute respiratory distress syndrome, with a strong inverse correlation with PaO₂/FiO₂ ratios, and showed that blocking CitH3 improved lung function and survival in mice [27]. This is supported by the fact that neutralizing CitH3 with monoclonal antibodies in murine LPS shock reduces cytokines, attenuates acute lung injury, and improves survival [5] Other studies involving adult and pediatric ARDS cohorts demonstrated elevated CitH3 levels in patients requiring ventilatory support. [28, 29].

The superior performance of CitH3 likely results from its direct relationship with NET formation and the acute immune response in septic shock. Meanwhile, PCT remains valuable and widely used, although evidence suggests it performs best when interpreted in conjunction with clinical scores or additional biomarkers.

This study has certain limitations that should be considered when interpreting the findings. First, generalizability may be limited by the small sample size and single-center design. Second, rather than being the result of pre-planned matching or stratification, the observed equal (50:50) male-to-female ratio across cohorts occurred coincidentally during random recruitment. This could introduce selection bias and may not accurately reflect the ICU population. Third, the lack of serial assessments prevented evaluation of temporal fluctuations in CitH3, and confounding variables such as the timing of antibiotics and non-infectious sources of systemic inflammation could have affected biomarker levels.

## Conclusion

CitH3 is a reliable biomarker of septic shock. It demonstrated superior diagnostic performance compared with PCT in diagnosing septic shock and showed strong correlations with clinical severity (SOFA score) and ICU length of stay. Our data indicate that CitH3 is a promising marker for predicting ventilation and mortality and could serve as a reliable candidate for routine integration into sepsis management protocols. Validation of the results of this study is recommended on a larger scale to define standardized clinical cutoffs and determine how CitH3 could mark a significant change in how septic shock is diagnosed and stratified, ultimately improving patient care and outcomes. The correlation between serum concentrations and the prognosis and severity of sepsis may provide important new insights into its pathophysiology.

## Data Availability

The datasets used and/or analyzed during the current study are available from the corresponding author on reasonable request.

## Abbreviation

ANC: Absolute Neutrophil Count
AUC: Area Under the Curve
CitH3: Citrullinated Histone H3
CBC: Complete Blood Count
COVID-19: Coronavirus Disease 2019
CRP: C-Reactive Protein
DAMP: Damage-Associated Molecular Pattern
DNA: Deoxyribonucleic Acid
ELISA: Enzyme-Linked Immunosorbent Assay
ICU: Intensive Care Unit
IQR: Interquartile Range
NET: Neutrophil Extracellular Trap(s)
NPV: Negative Predictive Value
PAD: Protein Arginine Deiminase
PCT: Procalcitonin
PPV: Positive Predictive Value
ROC: Receiver Operating Characteristic (curve)
SD: Standard Deviation
SOFA: Sequential Organ Failure Assessment
TLC: Total Leukocyte Count
